# The effects of environmental factors associated with childhood urbanicity on brain structure and cognition

**DOI:** 10.1186/s12888-023-05066-3

**Published:** 2023-08-17

**Authors:** Xiao Zhang, Hao Yan, Hao Yu, Yuyanan Zhang, Hao Yang Tan, Dai Zhang, Weihua Yue

**Affiliations:** 1 Institute of Mental Health, National Clinical Research Center for Mental Disorders, Peking University Sixth Hospital, 51 Huayuanbei Road, Haidian District, Beijing, 100191 China; 2 NHC Key Laboratory of Mental Health (Peking University), 51 Huayuanbei Road, Haidian District, Beijing, 100191 China; 3 Department of Psychiatry, Jining Medical University, Jining, Shandong China; 4 Lieber Institute for Brain Development, Baltimore, MD 21205 US; 5 Department of Psychiatry and Behavioral Sciences, Johns Hopkins University School of Medicine, Baltimore, MD 21205 US; 6 PKU-IDG/McGovern Institute for Brain Research of Peking University, &Chinese Institute for Brain Research, 51 Huayuanbei Road, Haidian District, Beijing, 100191 China; 7 Research Unit of Diagnosis and Treatment of Mood Cognitive Disorder, Chinese Academy of Medical Sciences, 51 Huayuanbei Road, Haidian District, Beijing, 100191 China

**Keywords:** Childhood environment, Urbanicity, Grey matter volume, Cognition, Having siblings, Maternal education

## Abstract

Urbanization is a trend lasting for more than one century worldwide. Four hundred ninety male and female adult Chinese Han participants with different urban and rural childhoods were included in this study. Early-life urban environment was found benefit for total grey matter volume (GMV), dorsolateral prefrontal cortex (DLPFC) GMV, temporal pole (TP) GMV and cognition function, and negatively correlated with medial prefrontal cortex (MPFC) GMV. Regression analysis showed that maternal education was a protective factor for total and DLPFC GMVs, while having siblings was better for MPFC GMV. Total, DLPFC and TP GMVs acts mediation effects between childhood urbanicity and different cognitive domains. These findings may suggest some pros and cons on brain structure associated with childhood urbanicity and related environmental factors.

**Supplementary Information:**

The online version contains supplementary material available at 10.1186/s12888-023-05066-3.

## Highlights

Urbanization is a trend lasting for more than one century, we did in-depth research which specific environment factor affects the brain structure and cognition function.

Early-life rural environment was benefit for the grey matter volume (GMV) of medial prefrontal cortex, having siblings was a protective factor for MPFC GMV.

Early-life urbanicity was benefit for cognition function and the GMV of total and dorsolateral prefrontal cortex, of which maternal education was a protective factor.

Total, DLPFC and TP GMVs acts mediation effects between childhood urbanicity and different cognitive domains.

**Supplementary Information:**

The online version contains supplementary material available at 10.1186/s12888-023-05066-3.

## Introduction

Urbanization has been progressing worldwide for over a century. In contrast to rural settings, cities attract people by offering opportunities in terms of income, education, health and social services [1]. Although urban areas may yield a higher quality of life on average, residing in cities may also have some negative impacts on physical well-being and mental health (World Health Organization and UN [2]. Previous studies in developed countries have shown that urban upbringing is an environmental risk factor for some psychiatric disorders. For example, the incidence and prevalence rates of schizophrenia seem to increase with increasing urbanicity [3, 4]. Studies in developed countries have shown that the gray matter volume (GMV) of the right dorsolateral prefrontal cortex is negatively correlated with urban upbringing [5]. In addition, in a social stress task study, researchers found that currently living in a city was associated with increased amygdala activity, whereas an urban upbringing affected the perigenual anterior cingulate cortex (pACC) [6]. These works suggest that the childhood residential environment might differentially influence the development of the brain, and early-life urbanicity is a risk factor for impaired brain development and mental health.

However, unlike the pastoral rural lifestyle observed in developed countries, inequalities between rural and urban settings as well as within urban areas have been persistent features in many developing countries [7, 8]. Moreover, inequalities in areas such as education resources, health care, housing and retirement benefits are observed in rural areas [9]. The differences in schizophrenia prevalence in China may reflect such issues; this pattern has shifted from higher prevalence in urban areas in the 1980s and 1990s [10, 11] to less apparent urban‒rural differences in recent years [12–15]. In fact, the prevalence of mental disorders was higher in rural communities than in urban communities in China in the most recent national survey [16]. A recent study found that urbanicity was associated with perspective-taking and depression symptoms, and this was mediated by neural variables [17]. While early-life urbanicity may have benefits, it is important to study the impacts of early-life urbanicity on brain development and mental health in developing countries, such as China.

China has undergone large-scale urbanization since the 1980s as well as unique economic development [18]. Its’ urbanization rate has risen from 15–20% of the population to 51% in 2011 [19]. This provides a unique opportunity to study the effect of childhood urbanization. We selected subjects whose current urbanicity conditions were similar, thus maximizing the effect of urbanicity on upbringing. In our previous report, we analyzed voxel-based morphometry (VBM) data after adjusting for the total GMV, similar to previous research [5]. It has been shown in previous work that the total GMV and MCCB scores were higher in subjects with greater early-life urbanicity [20]. However, including the total GMV as a covariate may affect the benefits of early-life urbanicity. Thus, we used a less strict standard to determine whether there are some positive influences on GMV of early-life urbanicity. Additionally, previous studies have reported that subjects raised in a rural environment had a larger GMV of the medial prefrontal cortex (MPFC) [20] but did not explore associated environmental factors, such as parental education and presence of siblings. Hence, we analyzed the brain structural data in greater detail, explored potential associated environmental factors in this study.

Some brain regions could have important effect on cognition related to individual development and social function. The dorsolateral prefrontal cortex (DLPFC) is a key cortical region plays an important role in executive function, such as working memory, cognitive flexibility, planning, inhibition and abstract reasoning [21]. The temporal pole is another interested region, it plays an important role in episodic memory, semantic memory [22] and visual perception processing system [23, 24]. In our previous study, local spontaneous brain activity (regional homogeneity, ReHo) mediated the influence of urbanicity on the speed of processing [25]. However, we do not know whether GMV mediates the relationship between urbanicity and cognition. Hence, we investigated the effects of GMV changes affected by childhood urbanicity on cognition using mediation analyses in this study.

## Methods and materials

### Participants

A total of 522 healthy subjects were recruited from the local community; of these, 32 were excluded due to incomplete residential information, low image quality or outliers in cognitive test scores. Thus, 490 subjects were finally included in the current analysis. All participants were assessed by experienced psychiatrists using the Structured Clinical Interview for DSM-IV-TR Axis I Disorders, Research Version, Non-Patient Edition (SCID-I/NP) to exclude any individuals with mental disorders. In addition, eligible subjects for our study had to meet the following criteria: (1) were aged 18 to 45 years, right-handed, Chinese Han ancestry; (2) had no history of neurological diseases or substance dependence; (3) had no history of more than 5 min of loss of consciousness; and (4) had no visible abnormalities on the MR images, confirmed by two experienced radiologists. We collected structural imaging, cognitive assessment and questionnaire data for all subjects. We recruited subjects currently living in Beijing for at least 1 year. All subjects included in our study had finished the nine-year compulsory education program in China. This study was approved by the local ethics committee. Written consent was obtained from each subject after description of the study. The detailed demographic information is listed in Table 1.

**Table 1:** Basic demographical characteristics of subjects

	Group A	Group B	Group C	Group D	F/χ^2^	p
Gender (male/female)	64/64	58/55	58/68	60/63	0.74	0.86
Age (months)	315.20 (36.40)	295.16 (44.66)	293.96 (46.78)	289.95 (48.69)	8.23	< 0.001
Education (years)	17.34 (2.47)	16.58 (2.74)	16.63 (2.24)	16.63 (2.41)	2.78	0.040
Height	167.17 (7.31)	168.20 (7.94)	168.90 (7.87)	169.64 (8.40)	2.24	0.083
Weight	61.21 (11.40)	61.51 (11.63)	60.53 (11.66)	64.35 (14.11)	2.34	0.073
Father Age at Birth	27.19 (6.22)	26.82 (5.78)	27.54 (4.97)	29.37 (4.31)	5.34	0.001
Father Education (years)	8.95 (3.05)	10.30 (3.41)	11.79 (3.45)	14.11 (3.32)	53.53	< 0.001
Mother Age at Birth	26.09 (4.73)	25.73 (5.46)	25.86 (4.03)	27.42 (3.47)	3.71	0.012
Mother Education (years)	7.23 (3.72)	8.61 (3.94)	11.13 (3.44)	13.75 (3.17)	80.14	< 0.001
Parents’ Marriage (Normal/Other)	110/18	103/10	108/18	99/24	5.48	0.14
Single Child (Yes/No)	17/111	35/78	66/60	110/13	160.59	< 0.001
MCCB T Score	50.45 (4.67)	51.68 (5.08)	53.19 (4.16)	55.11 (4.50)	23.71	< 0.001
Total GMV	732.92 (57.51)	746.14 (65.44)	748.81 (57.07)	755.54 (66.52)	2.99	0.031

*MCCB* MATRICS (Measurement and Treatment Research to Improve Cognition in Schizophrenia) Consensus Cognitive Battery, *GMV* Grey Matter Volume

To determine urbanicity, subjects provided details regarding their places of residence from birth to the present (More detailed information descripted in supplementary materials). Using the local population size [26] as the standard and Chinese administrative divisions as the supplement, we defined rural areas as agricultural regions with populations typically < 10,000; urban areas were defined as cities with populations typically more than 100,000 (and often well over several million). In the main text, we divided the subjects into 4 groups according to urbanicity from low to high: individuals who were born in and lived in rural areas for > 18 years since birth (Group A, *N* = 128), those who lived in rural areas between birth and the age of 18 years (Group B, *N* = 113), those who lived in cities since before the age of 12 years (Group C, *N* = 126), and those who were born in and continued to live in cities (Group D, *N* = 123). We also tried to evaluate childhood urbanicity with an urbanicity score (similar to that used in previous studies) and dividing subjects into 2 groups to see if the findings were robust. The urbanicity score was defined according to population size as follows: population < 10,000 = 1, less than 1,000,000 = 2, and more than 1,000,000 = 3; the category scores were then multiplied by the number of years spent in that location until the age of 15 years.

### Data collection

All subjects were scanned with a 3.0 T GE Discovery MR750 scanner. Before scanning, all subjects were instructed to move as little as possible. Foam pads were used to minimize head motion. T1-weighted high-resolution structural images were acquired in a sagittal orientation using an axial 3D fast, spoiled gradient recalled (FSPGR) sequence with the following parameters: time of repetition (TR) = 6.66 ms, time of echo (TE) = 2.93 ms, field of view (FOV) = 256 × 256 mm^2^, slice thickness/gap = 1.0/0 mm, acquisition voxel size = 1 × 1 × 1 mm^3^, flip angle = 12°, and 192 contiguous sagittal slices.

To identify potential factors that may influence gray matter volume, we examined age, sex, years of education, height, weight, and the presence of siblings. We also collected information on the subjects’ parents, including years of education, age at childbearing, and marital status. Regarding cognitive performance, we used the MATRICS (Measurement and Treatment Research to Improve Cognition in Schizophrenia) Consensus Cognitive Battery (MCCB) and calculated the T score using the official software [27–29].

### Structural MRI data analysis

Structural images were processed using DPABI [30], a MATLAB toolbox that uses SPM (http://www.fil.ion.ucl.ac.uk/spm) and the new segment function of DARTEL [31]. DARTEL is believed to have better segmentation results than classic methods [32]. Briefly, image processing included the following steps: (1) transformation of structural images into NIFTI format; (2) reorientation of structural images such that the millimeter coordinates of the anterior commissure (AC) matched the origin [000]; (3) segmentation of T1-weighted MR images into gray matter, white matter, cerebrospinal fluid and three other noncerebral tissue classes as well as normalization to Montreal Neurological Institute (MNI) space with a diffeomorphic image registration algorithm (DARTEL); (4) normalization of the whole-brain images of individual participants to the SPM default mask and modulation of GMV; and (5) smoothing of the segmented, normalized and modulated GM images with an 8-mm full width at half maximum isotropic Gaussian kernel.

**Fig. 1:**
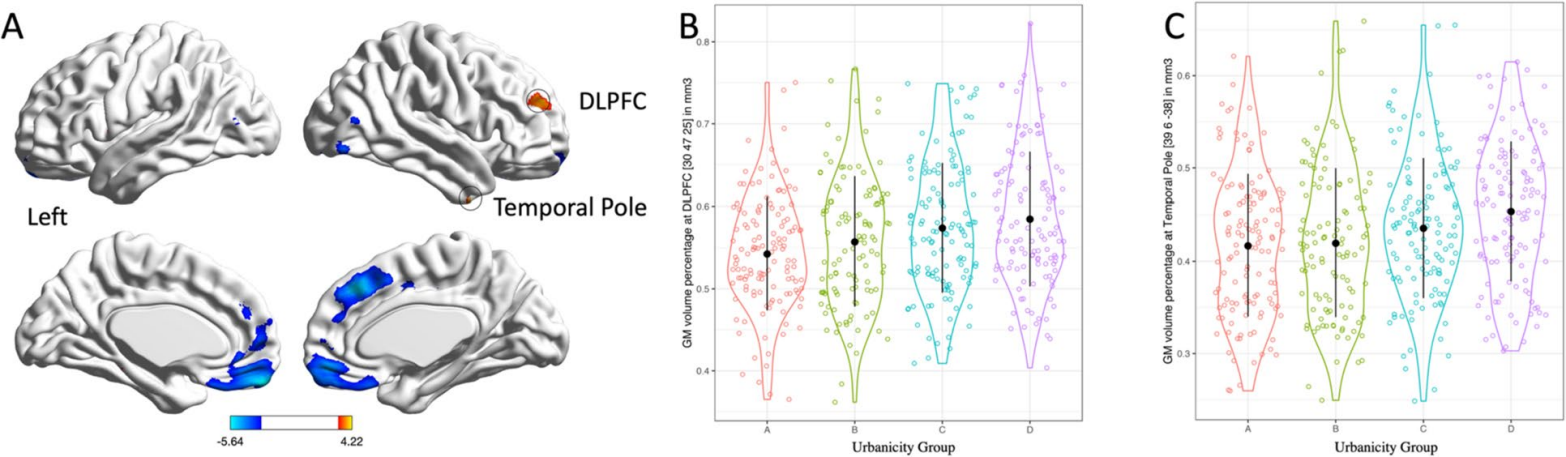
Relationship between early-life urbanicity and brain GMV. **A** T map of the correlation between early-life urbanicity and GMV (red: urban > rural, blue: rural > urban, shown at *p* < 0.001 uncorrected). **B** Scatterplot of urban > rural findings regarding the GMV of Brodmann area 10 in the DLPFC (peak atlas = [30,47,25], T = 4.22, cluster size = 345, *p* < 0.001 uncorrected). **C** Scatterplot of urban > rural findings regarding the GMV of Brodmann area 36 in the temporal pole (peak atlas = [39, 6, -38], T = 4.09, cluster size = 217, *p* < 0.001 uncorrected)

The effect of early-life urbanicity on GMV was tested in a general linear model (Group A: -3, Group B: -1, Group C: 1, Group D 3) with age, age^2^ [33] sex, years of education, the total GMV and MCCB scores included as nuisance covariates. Since the total GMV and MCCB scores were higher in subjects with greater early-life urbanicity, this analysis may overlook some positive influence of early-life urbanicity on GMV. To identify the benefits of early-life urbanicity, we attempted to uncover overlooked results using the following thresholds (*p* < 0.001 uncorrected, Fig. 1). Similar results (see Supplementary Figures S 1 and S 2) were obtained when using different methods to define childhood urbanicity.

### Regression analysis

To understand which environmental factors, including urbanicity, caused the GMV change, we performed multiple linear regression with the total GMV and GMVs of regions of interest (ROIs) (urban > rural peak, rural > urban peak) as dependent variables with a backward method. For the total GMV model, the predictor variables were the questionnaire data described above and urbanicity (A: -3, B: -1, C: 1, D: 3). Since the total GMV could affect the ROI GMVs and be related to height and weight, we replaced height and weight with the total GMV for the ROI GMV models while all other predictor variables were the same. In addition, using the total GMV as one predictor for ROI GMVs allowed us to test whether other environmental factors affected ROI GMVs after removing the influence of the total GMV.

### Mediation analysis

After determining the differences in cognition according to urban and rural childhoods, we were interested in the impact of these brain regions of interest on cognitive performance. Therefore, to study whether GMV changes influenced the relationship between urbanicity and MCCB scores, we used R software (https://www.r-project.org/) to analyze the mediating effect of GMV changes, and applied corrections for multiple comparison since we analyzed five GMV results. The first step was to construct the regression model in which MCCB score (Y) was the dependent factor and urbanicity (X) was the independent factor (Y = cX + e_1_). The second step was to construct the regression model in which ROI GMVs (M) was the dependent factor and urbanicity (X) was the independent factor (M = aX + e_2_). The third step was to construct the regression model where Y was the dependent factor and X and M were the independent factors (Y = c’X + bM + e_3_). Then, we performed a Sobel test and calculated the indirect effect of each model (Table 3). The detailed procedure can be found in this article [34].

## Results

### Demographic and cognitive characteristics of the sample

The four groups of subjects were matched in terms of sex; Group A (mostly rural) had a slightly increased age (21 ~ 25.71 months) and education level (0.68 ~ 0.78 years). In addition, the proportion of only children significantly increased in the same direction as early-life urbanicity. In addition, greater early-life urbanicity was associated with more parental education. Parental ages also significantly differed among the 4 groups, with the most urban group having the highest mean age of parents.

Regarding cognition, all groups had more than 16 years of education on average, which means nearly all subjects finished college and some amount of graduate school. It was therefore expected that their MCCB T scores would exceed 50. Nevertheless, subjects with rural childhoods had a significantly lower overall score. Detailed information is included in Table 1.

### Main effect of urban upbringing on GMV

In previous work, a negative correlation was found between early-life urbanicity and the MPFC GMV in terms of Brodmann area (BA) 11 (x = -3, y = 56, z = -21; T = 5.64; *p* < 0.05 FWE corrected) and BA 8 (x = 6, y = 33, z = 40; T = 5.48; *p* < 0.05 FWE corrected) [20]. In this article, the adulthood brain GMVs positively correlated with early-life urbanicity mainly locate at the right dorsolateral prefrontal cortex (DLPFC) GMV in BA10 (x = 30, y = 47, z = 25; T = 4.22; cluster size = 345; *p* < 0.001, uncorrected) and the right temporal pole GMV in BA36 (x = 39, y = 6, z = -38; T = 4.09; cluster size = 217; *p* < 0.001, uncorrected, Fig. 1). Other correlated clusters are relatively small and T values are relatively low (See Supplementary Table S1, *p* < 0.001, uncorrected).

### Factors that may influence the GMVs of the DLPFC and MPFC

**Fig. 2:**
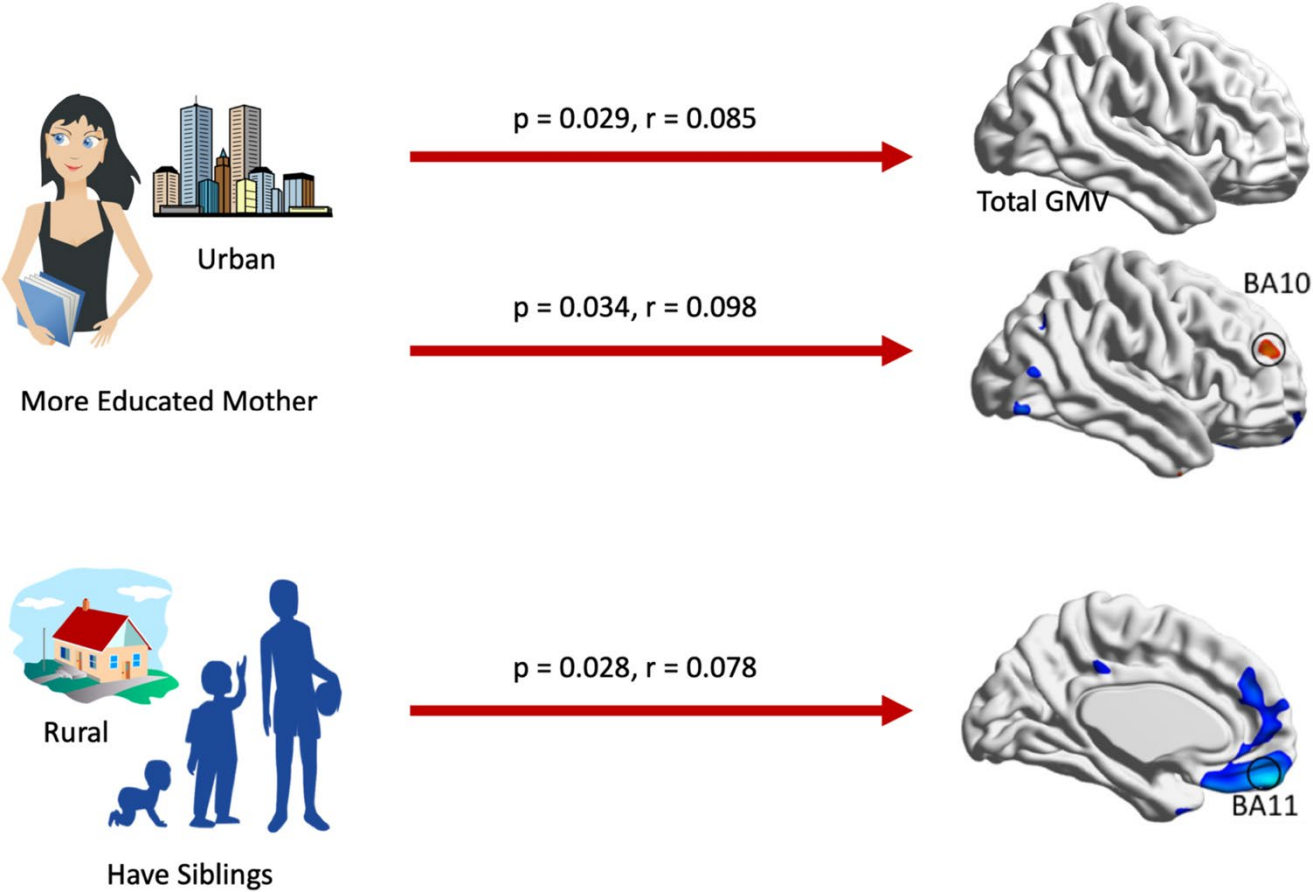
Key factors of rural and urban environments that may influence brain structure in adulthood. Regression analysis showed that the presence of siblings was a protective environmental factor for MPFC GMV, and rural subjects have more presence of siblings. Similarly, higher maternal education was a protective environmental factor for the total GMV and DLPFC GMV, and urban subjects have higher maternal education

In the multiple linear regression model, we included the result with the highest R^2^ value. For models with the same R^2^ value, we displayed the model with the fewest independent factors. In the total GMV model (Table 2), the total GMV was significantly affected by sex (greater in males), age (greater in younger individuals), weight (greater with heavier weights), and maternal years of education (greater with higher maternal education). In addition to the above environmental factors and urbanicity, the presence of siblings had a significant positive influence on the GMV of BA11, and maternal years of education showed a significant positive influence on the GMV of the DLPFC (Table 2, Fig. 2).

**Table 2:** Multiple linear regression model of total GMV and interested regional interested GMVs using environment variables as predictor

	Unstandardized Coefficients	Sig	95% Confidence Interval for B		Standardized Coefficients	
	B	Std Error	Lower Bound		Upper Bound	Beta
**Total GMV: Adjusted R**^**2**^**: 0.547; F Sig.: < 0.001; Std. Error of the Estimate: 41.72; N: 485**						
(Constant)	697.489	74.099	< 0.001	551.888	843.09	
Age	-0.239	0.047	< 0.001	-0.331	-0.146	-0.174
Gender	-66.432	6.043	< 0.001	-78.306	-54.558	-0.536
Education Year	1.223	0.828	0.140	-0.404	2.851	0.049
Height	0.647	0.419	0.123	-0.176	1.47	0.083
Weight	1.042	0.237	< 0.001	0.577	1.507	0.207
**Mother Education**	1.216	0.556	0.029	0.123	2.31	0.085
Having Siblings	8.75	4.848	0.072	-0.776	18.277	0.07
Urbanicity	2.149	1.11	0.053	-0.032	4.33	0.078
**Temporal Pole: Adjusted R**^**2**^**: 0.226; F Sig.: < 0.001; Std. Error of the Estimate: 0.06856; N: 485**						
(Constant)	0.052	0.043	0.222	-0.032	0.137	
Mother Education	-0.001	0.001	0.16	-0.003	0.001	-0.07
Single Child	-0.012	0.008	0.123	-0.027	0.003	-0.077
Total GMV	0.001	0	< 0.001	0	0.001	0.438
Urbanicity	0.004	0.002	0.023	0.001	0.008	0.12
**DLPFC: Adjusted R**^**2**^**: 0.531; F Sig.: < 0.001; Std. Error of the Estimate: 0.05372; N: 485**						
(Constant)	-0.058	0.038	0.132	-0.133	0.017	
Age	0	0	0.002	0	0	-0.1
Father Education	-0.002	0.001	0.054	-0.003	0	-0.085
**Mother Education**	0.002	0.001	0.034	0	0.003	0.098
Having Siblings	0.011	0.006	0.089	-0.002	0.023	0.067
Total GMV	0.001	0	< 0.001	0.001	0.001	0.699
Urbanicity	0.004	0.001	0.004	0.001	0.007	0.12
**BA11: Adjusted R**^**2**^**: 0.592; F Sig.: < 0.001; Std. Error of the Estimate: 0.03899; N: 485**						
(Constant)	-0.169	0.041	< 0.001	-0.249	-0.09	
Age	-7.23E-05	0	0.08	0	0	-0.054
Gender	0.015	0.005	0.003	0.005	0.025	0.124
**Having Siblings**	0.01	0.004	0.028	0.001	0.018	0.078
Total GMV	0.001	0	< 0.001	0.001	0.001	0.852
Urbanicity	-0.005	0.001	< 0.001	-0.007	-0.003	-0.172
**BA8: Adjusted R**^**2**^**: 0.517; F Sig.: < 0.001; Std. Error of the Estimate: 0.04526; N: 485**						
(Constant)	-0.141	0.047	0.003	-0.234	-0.049	
Age	-7.56E-05	0	0.111	0	0	-0.053
Gender	0.015	0.006	0.009	0.004	0.027	0.118
Total GMV	0.001	0	< 0.001	0.001	0.001	0.795
Urbanicity	-0.006	0.001	< 0.001	-0.008	-0.004	-0.208

*GMV* Grey Matter Volume, *DLPFC* Dorsolateral prefrontal cortex, *BA* Brodmann area

### Mediating effects of altered ROI GMVs

**Fig. 3:**
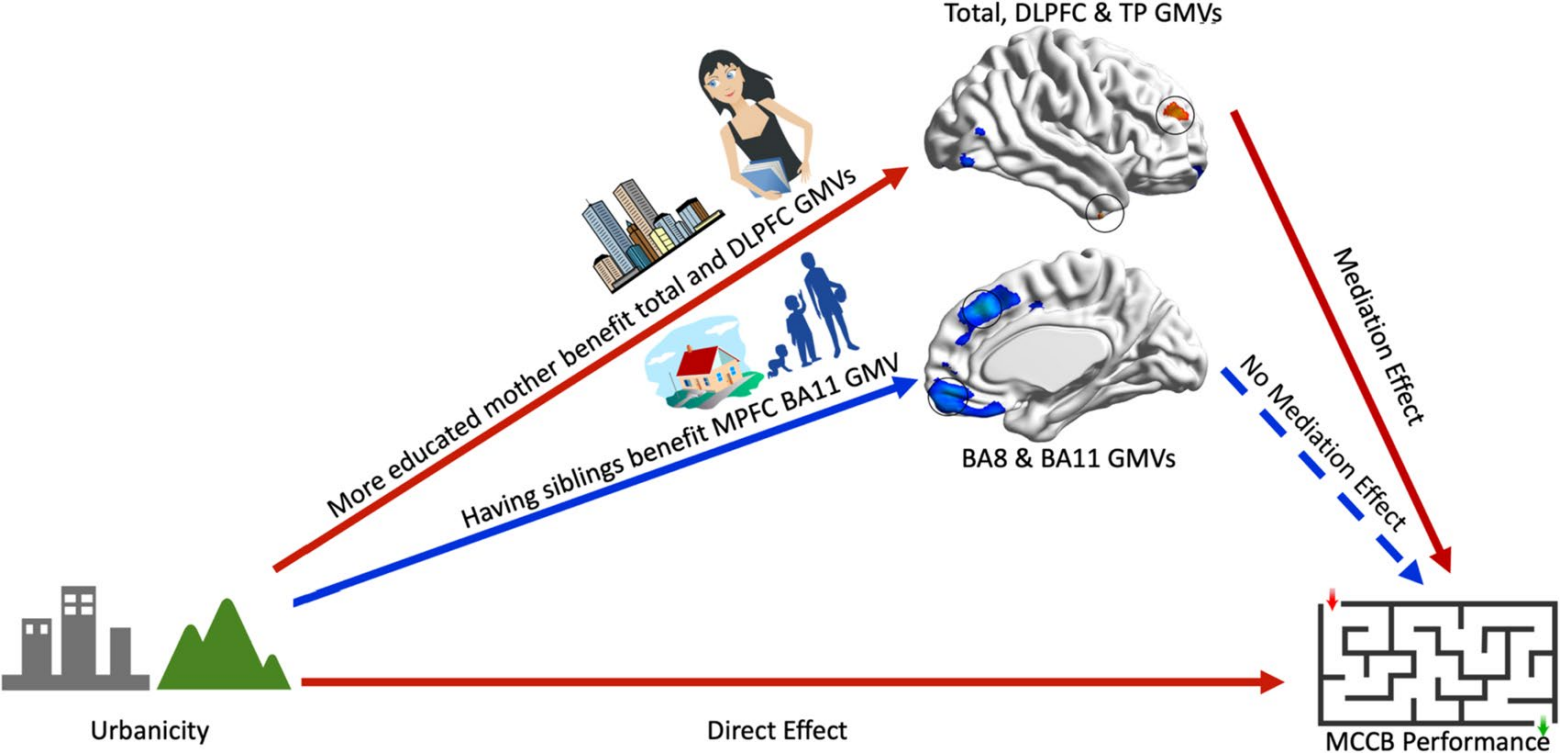
A schematic overview of the study findings. In this study, we found that an early-life urban environment benefitted brain development in terms of the total, dorsolateral prefrontal cortex (DLPFC), and temporal gray matter volumes (GMVs), while an early-life rural environment benefitted the GMV of the medial prefrontal cortex (MPFC) in Brodmann area (BA) 8 and BA11. Regression analysis showed that having siblings is a protective environment factor for MPFC BA11 GMV; and higher mother’s education is a protective environment factor for total and DLPFC GMV. Mediation analysis showed that the total, DLPFC and temporal pole GMVs, which reflect the benefits of early-life urbanicity, could mediate the relationship between early-life urbanicity and cognitive function in adulthood

Since there were increases in the cognitive scores and total GMV with greater early-life urbanization, we further studied the mediating effect of altered ROI GMVs in the relationship between early-life urbanicity and adulthood cognitive function (Table 3, Fig. 3). We found that the total GMV, DLPFC GMV, and temporal pole GMV mediated the relationship of urbanicity with reasoning and problem solving. In addition, the total GMV and DLPFC GMV mediated the relationship between urbanicity and working memory. Finally, the DLPFC GMV mediated the relationship between urbanicity and the MCCB total score. These mediation effects survived Bonferroni correction for multiple comparisons. For the rural > urban brain regions, there was no mediating effect on the relationship between urbanicity and MCCB scores at the above threshold.

**Table 3:** Mediation analysis of GMVs (M) on Urbanicity (X) and MCCB performance (Y)

Variables	Step 1 c	Step 1	Step 3 b	Step 3	Step 3 c’	Step 3	Sobel	Sobel	Indirect	Result
	***p* value**	**c**	***p* value**	**b**	***p* value**	**c’**	***p* value**	**z value**	**Effect**	
**Step 2 (M: total GMV) M = 3.61X + 745.79, *p* = 1.73E-02**										
Attention/Vigilance	1.83E-03	0.568	7.05E-01	5.927	4.22E-03	0.549	9.30E-01	0.562	0.019	N.M
Working Memory	4.39E-03	0.644	6.25E-05	19.326	2.67E-02	0.527	7.25E-02	2.448	0.117	M
Verbal Learning	6.85E-10	1.215	1.47E + 00	7.785	4.12E-10	1.212	1.62E + 00	0.089	0.004	N.M
Visual Learning	2.30E-03	0.433	1.73E + 00	-2.317	1.62E-03	0.470	1.84E + 00	-1.362	-0.037	N.M
Reasoning and Problem Solving	8.90E-09	1.042	2.27E-05	21.823	1.85E-07	0.925	6.55E-02	2.688	0.118	M
Social cognition	3.97E-01	0.296	1.49E + 00	-0.957	3.02E-01	0.317	1.63E + 00	-0.577	-0.021	N.M
Speed of Processing	5.60E-10	1.088	1.90E + 00	0.453	1.64E-09	1.134	2.00E + 00	-1.263	-0.046	N.M
Total MCCB Score	8.00E-15	0.758	1.01E-01	7.424	9.50E-14	0.737	3.39E-01	1.071	0.022	N.M
**Step 2 (M: DLPFC) M = 7.23E-03X + 0.56, *p* = 1.59E-05**										
Attention/Vigilance	1.83E-03	0.568	1.00E + 00	5.927	3.76E-03	0.549	2.87E + 00	0.562	0.019	N.M
Working Memory	4.39E-03	0.644	2.70E-03	19.326	3.58E-02	0.527	7.18E-02	2.448	0.117	M
Verbal Learning	6.83E-10	1.215	8.13E-01	7.785	1.92E-09	1.212	4.65E + 00	0.089	0.004	N.M
Visual Learning	2.30E-03	0.433	2.59E + 00	-2.317	9.89E-04	0.470	8.65E-01	-1.362	-0.037	N.M
Reasoning and Problem Solving	8.90E-09	1.042	7.59E-05	21.823	5.96E-07	0.925	3.59E-02	2.688	0.118	M
Social Cognition	3.97E-01	0.296	4.22E + 00	-0.957	3.32E-01	0.317	2.82E + 00	-0.577	-0.021	N.M
Speed of Processing	5.59E-10	1.088	4.64E + 00	0.453	2.44E-10	1.134	1.03E + 00	-1.263	-0.046	N.M
Total MCCB Score	7.98E-15	0.758	4.31E-02	7.424	1.52E-13	0.737	1.42E + 00	1.071	0.022	M
**Step 2 (M: Temporal Pole) M = 6.23E-03X + 0.43, *p* = 2.79E-4**										
Attention/Vigilance	1.83E-03	0.568	1.52E + 00	-4.775	6.40E-04	0.621	5.32E-01	-1.615	-0.053	N.M
Working Memory	4.39E-03	0.644	3.69E-01	10.092	1.12E-02	0.601	1.21E + 00	1.169	0.043	N.M
Verbal Learning	6.85E-10	1.215	3.60E + 00	2.015	5.35E-10	1.243	2.09E + 00	-0.810	-0.028	N.M
Visual Learning	2.30E-03	0.433	3.74E + 00	-1.162	1.47E-03	0.455	1.70E + 00	-0.953	-0.022	N.M
Reasoning and Problem Solving	8.90E-09	1.042	2.16E-02	14.570	9.95E-08	0.984	4.42E-01	1.704	0.059	M
Social Cognition	3.97E-01	0.296	1.14E + 00	-5.907	2.24E-01	0.343	7.35E-01	-1.450	-0.048	N.M
Speed of Processing	5.60E-10	1.088	4.01E + 00	-1.258	1.97E-10	1.133	8.19E-01	-1.392	-0.045	N.M
Total MCCB Score	8.00E-15	0.758	2.50E + 00	1.921	7.85E-15	0.772	2.20E + 00	-0.771	-0.013	N.M
**Step 2 (M: BA11) M = -2.58E-03X + 0.48, *p* = 2.32E-1**										
Attention/Vigilance	1.83E-03	0.568	1.09E + 00	6.848	1.12E-03	0.590	1.12E + 00	-1.218	-0.022	N.M
Working Memory	4.39E-03	0.644	1.06E-04	28.518	7.35E-04	0.724	3.31E-01	-1.837	-0.080	N.M
Verbal Learning	6.85E-10	1.215	4.65E + 00	-0.598	6.30E-10	1.223	3.14E + 00	-0.485	-0.008	N.M
Visual Learning	2.30E-03	0.433	6.78E-01	-6.446	3.56E-03	0.420	1.52E + 00	1.028	0.013	N.M
Reasoning and Problem Solving	8.90E-09	1.042	3.06E-02	16.758	1.09E-09	1.094	4.21E-01	-1.727	-0.052	N.M
Social Cognition	3.97E-01	0.296	2.76E-01	-11.220	5.55E-01	0.269	9.33E-01	1.321	0.027	N.M
Speed of Processing	5.60E-10	1.088	4.34E + 00	-0.991	5.40E-10	1.095	3.40E + 00	-0.413	-0.006	N.M
Total MCCB Score	8.00E-15	0.758	8.66E-01	4.651	1.85E-15	0.777	6.91E-01	-1.483	-0.018	N.M
**Step 2 (M: BA8) M = -2.42E-03X + 0.47, *p* = 2.35E-1**										
Attention/Vigilance	1.83E-03	0.568	3.92E + 00	1.639	1.59E-03	0.576	2.83E + 00	-0.575	-0.009	N.M
Working Memory	4.39E-03	0.644	4.87E-02	18.608	1.60E-03	0.695	4.96E-01	-1.649	-0.051	N.M
Verbal Learning	6.83E-10	1.215	2.06E + 00	-5.893	9.71E-10	1.211	3.95E + 00	0.267	0.005	N.M
Visual Learning	2.30E-03	0.433	4.39E + 00	-0.704	2.32E-03	0.435	4.36E + 00	-0.162	-0.002	N.M
Reasoning and Problem Solving	8.90E-09	1.042	1.63E-01	13.953	1.81E-09	1.085	5.25E-01	-1.621	-0.042	N.M
Social Cognition	3.97E-01	0.296	4.71E + 00	-0.456	3.98E-01	0.297	4.67E + 00	-0.082	-0.001	N.M
Speed of Processing	5.59E-10	1.088	1.20E + 00	-7.498	9.60E-10	1.079	2.73E + 00	0.605	0.009	N.M
Total MCCB Score	7.98E-15	0.758	2.19E + 00	2.831	3.29E-15	0.771	1.08E + 00	-1.236	-0.013	N.M

The *p* values in this table are shown after multiple comparison corrections. M. means mediation effect. N.M. means no mediation effect. Step 1 regression analysis is Y = cX + e_1_. Step 2 regression analysis is M = aX + e_2_. Step 3 regression analysis is Y = c’X + bM + e_3_. Then, we did Sobel test and calculated the indirect effect of each model

*MCCB* MATRICS Consensus Cognitive Battery, *GMV* Grey Matter Volume, *DLPFC* Dorsolateral prefrontal cortex, *BA* Brodmann area

## Discussion

We have expanded earlier finding of an increased total GMV & cognition with more early-life urbanicity, and increased MPFC GMV with more early-life rural experience [20]. We found a positive relationship between early-life urbanicity and DLPFC and TP GMVs in this study, revealing some benefits of early-life urbanicity. Regarding specific environmental factors, we found that having siblings was associated with increased MPFC GMV and that higher maternal education was associated with higher total and DLPFC GMVs. In addition, the total, MPFC and TP GMVs exerted a positive mediating effect on the relationship between early-life urbanicity and cognitive performance in adulthood, specifically in terms of working memory, reasoning and problem solving (Fig. 3).

The MPFC GMV appears to increase with early-life rural experiences, especially the GMV of the medial regions (BA11 and BA8). The direction of the early-life upbringing effect is consistent with that of a similar study in Germany [5]. The MPFC is believed to play an important role in social cognition [35]. In our sample, rural subjects had more siblings, and the presence of siblings was significantly associated with the GMV of BA11. Siblings may provide more peer companionship and social interactions in childhood, which may result in lower neural sensitivity to social stress [36]. Our Previous study found that lower MPFC activity in response to social status threat was correlated with higher trait anxiety and depression in subjects with urban childhoods but not in those with rural childhoods [20]. Our current findings suggest that the presence of siblings may be a protective environmental factor for MPFC function. Decreased GMV of the MPFC was observed in subjects with prior experiences of adversity and stress, which is considered a risk factor for psychiatric disorders [37]. In our sample, there was no mediating effect of the altered GMV in the MPFC (rural > urban) on the relationship between childhood urbanicity and cognition scores. Our findings suggest that rural subjects have similar educational and vocational achievement not through enhanced cognitive performance but rather through increased social resilience, since the main function of the MPFC is social cognition and decision-making [37].

In addition to previous positive effects of early-life urbanicity on the total GMV and cognitive performance, we identified larger DLPFC and temporal pole GMVs with more early-life urbanicity. We found that maternal education positively affected the total and DLPFC GMVs, which is consistent with the importance of maternal education for children’s outcomes [38, 39]. Maternal education has been associated with socioeconomic status [40] and could affect the quality of cognitive stimulation in the home, such as mother–child interactions, the availability of books, computers, trips, parental communication and so on [41, 42]. The meta-analysis showed that activation of the lateral DLPFC is associated with working memory and episodic memory, which are two key features of cognition [43]. The mediating effect of the DLPFC GMV on the relationship of early-life urbanicity with the MCCB total score (including working memory, reasoning and problem solving) adds to our knowledge about the function of this brain region. Previous studies have shown that the functions of the DLPFC include branching and recollection of attention [44]. Our finding validates this hypothesis since the process of solving mazes required subjects to keep the goal in mind while exploring. In a study on patients with Alzheimer's disease, changes in the volume of the temporal pole were related to cognitive impairment [45, 46]. We found that the temporal pole GMV mediated the relationship between childhood urbanicity and reason and problem solving, which suggests that it is important for cognition.

The GMV and cognition differences between individuals with urban and rural childhoods may reflect nutritional and socioeconomic status differences. Although there was a fourfold improvement in per capita consumption from 1990 to 2009, the period in which most of our subjects were children or adolescents [47], the urban‒rural income gap increased simultaneously, with urban incomes rising markedly relative to rural incomes [48]. There are studies showing that family income and poverty status are powerful correlates of the cognitive development and behavior of children, even after accounting for other differences such as maternal education [49]. Studies have shown that approximately 39% of infants and toddlers (ages 0 to 3 years) born and raised in rural Chinese villages exhibit cognitive or psychomotor delays [50]. It seems that this pattern may be present even in those with educational achievements similar to those of their urban peers. However, these conclusions are speculative since we did not directly assess early-life stress and family income, although we inferred family income and early-life stress according to parental education and social development patterns. With the comprehensive poverty alleviation efforts implemented in China, this effect may weaken in the future.

This study has several limitations. First, the most rural group of subjects was slightly older and had more years of education than the other groups of subjects. Although we controlled for age and years of education during the analysis, there may have been residual influences on the results. Second, we recruited healthy individuals, and the influence of the early-life environment may differ in patients with mental disorders. Third, since we recruited relatively highly educated people currently living in Beijing, our sample is not representative of people with low education levels and living in rural areas. Forth, although we tried our best to include all objective factors we could obtain, the factors we included might not sufficient to cover all the aspects of the complex childhood environment compounds. Further research needs to be done in more diverse samples from clinical and nonclinical populations and on additional variables that could directly reflect early-life urbanicity.

## Conclusion

In conclusion, we found a positive correlation of early-life urbanicity with cognitive scores and the total, DLPFC and TP GMVs and a negative correlation with the MPFC GMV. The increased total, DLPFC and TP GMVs partly mediated the relationship between early-life urbanicity and cognitive scores. In addition, the presence of siblings among individuals was associated with higher MPFC GMV, while maternal education was associated with better outcomes of total and DLPFC GMVs. On the one hand, this study replicates previous findings regarding the benefits of rural childhoods; on the other hand, this study suggests that there are also benefits from urban childhoods regarding brain and cognitive function.

### Supplementary Information

**Additional file 1: Supplement Method:** More detailed information about participants. **Figure S1.** Early-life urbanicity (2 groups) and GMV differences. **Figure S2.** Early-life urbanicity (urbanicity score) and GMV differences. **Table S1.** Regional GMVs that urban subjects are larger than rural subjects.

## Data Availability

The data and materials that support the findings of this study are available from the corresponding author upon reasonable request.
