# Supplementary material for: The effects of environmental factors associated with childhood urbanicity on brain structure and cognition
Source: BMC Psychiatry. 2023 Aug 17;23:598. doi: 10.1186/s12888-023-05066-3 (PMC10433654; doi:10.1186/s12888-023-05066-3)
Supplement: Supplementary file 1 — Additional file 1: Supplement Method: More detailed information about participants. Figure S1. Early-life urbanicity (2 groups) and GMV differences. Figure S2. Early-life urbanicity (urbanicity score) and GMV differences. Table S1. Regional GMVs that urban subjects are larger than rural subjects. [file 12888_2023_5066_MOESM1_ESM.docx]

# Supplement Materials

# Supplement Method: Participants

In our study, urbanicity was quantified using inhabitants number (Ministry of Housing and Urban-Rural Development 2014) with Chinese administrative divisions as supplement. The structural hierarchy of the administrative divisions of China includes provincial, prefectural, county and township level. The seat of the provincial, prefectural and county level government is considered as cities in national census. Township level is the basic level of political divisions in China, and towns usually have less than 10,000 residence inhabitants with several jurisdictional villages surrounded.

In our study, we define rural environment as administrative towns and villages usually less than 10,000 residence inhabitants. We specifically confirmed with participants that they lived in rural environments with sufficient green space and were predominantly engaged in agricultural cultivation. Currently, all subjects are living in Beijing, which is a super city with more than 20,000,000 inhabitants, and their educational and occupational achievements are similar. We specifically confirmed with participants that they are located in a typical urban environment, basically located within Beijing's main urban area (the fifth ring road), surrounded by modern urban buildings.

## Figure S1 Early-life urbanicity (2 groups) and GMV differences.


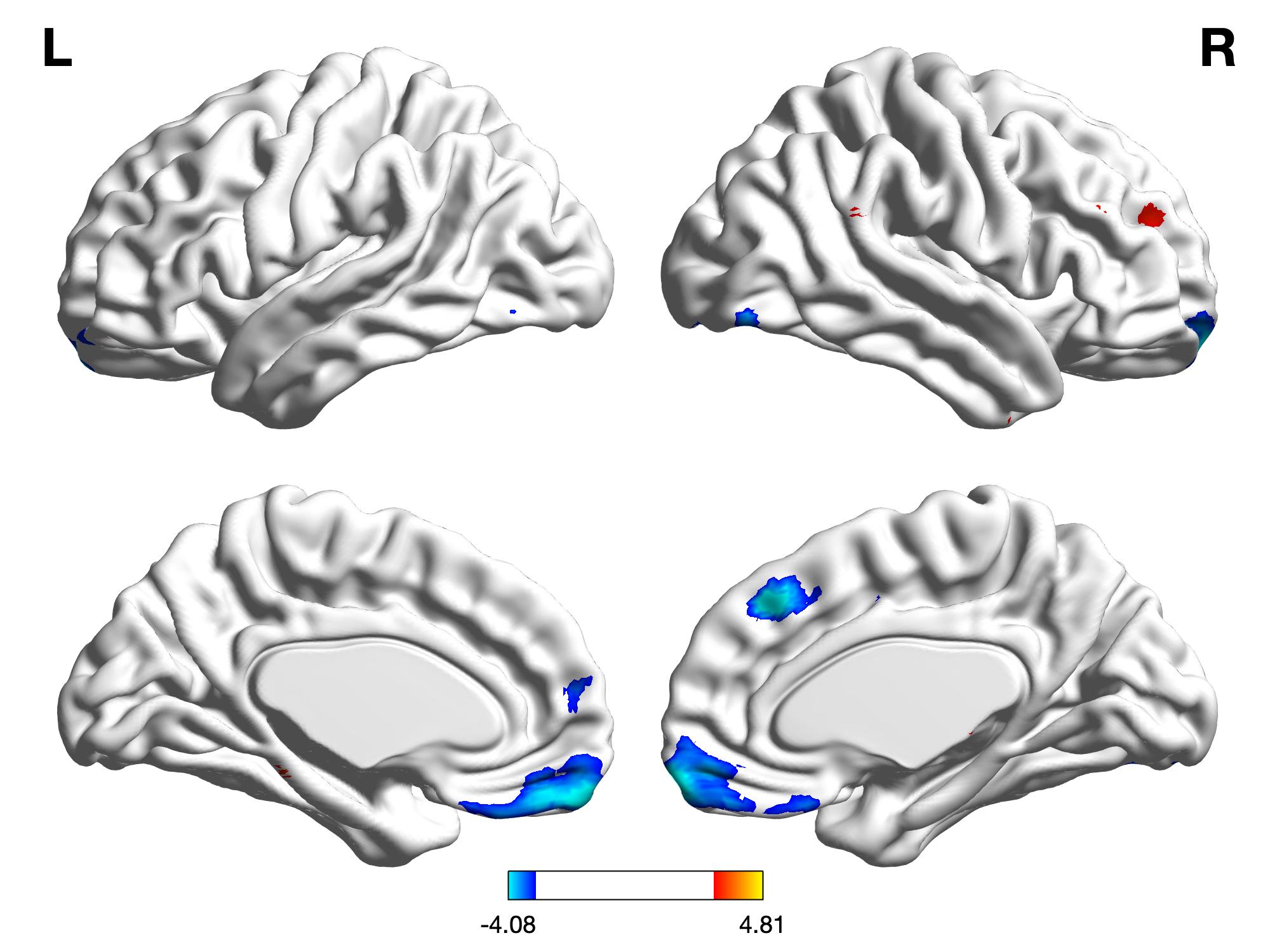


T-map of the correlation with early-life urbanicity and GMV (red means urban > rural, blue means rural > urban, shown at p<0.001 uncorrected).

## Figure S2 Early-life urbanicity (urbanicity score) and GMV differences.


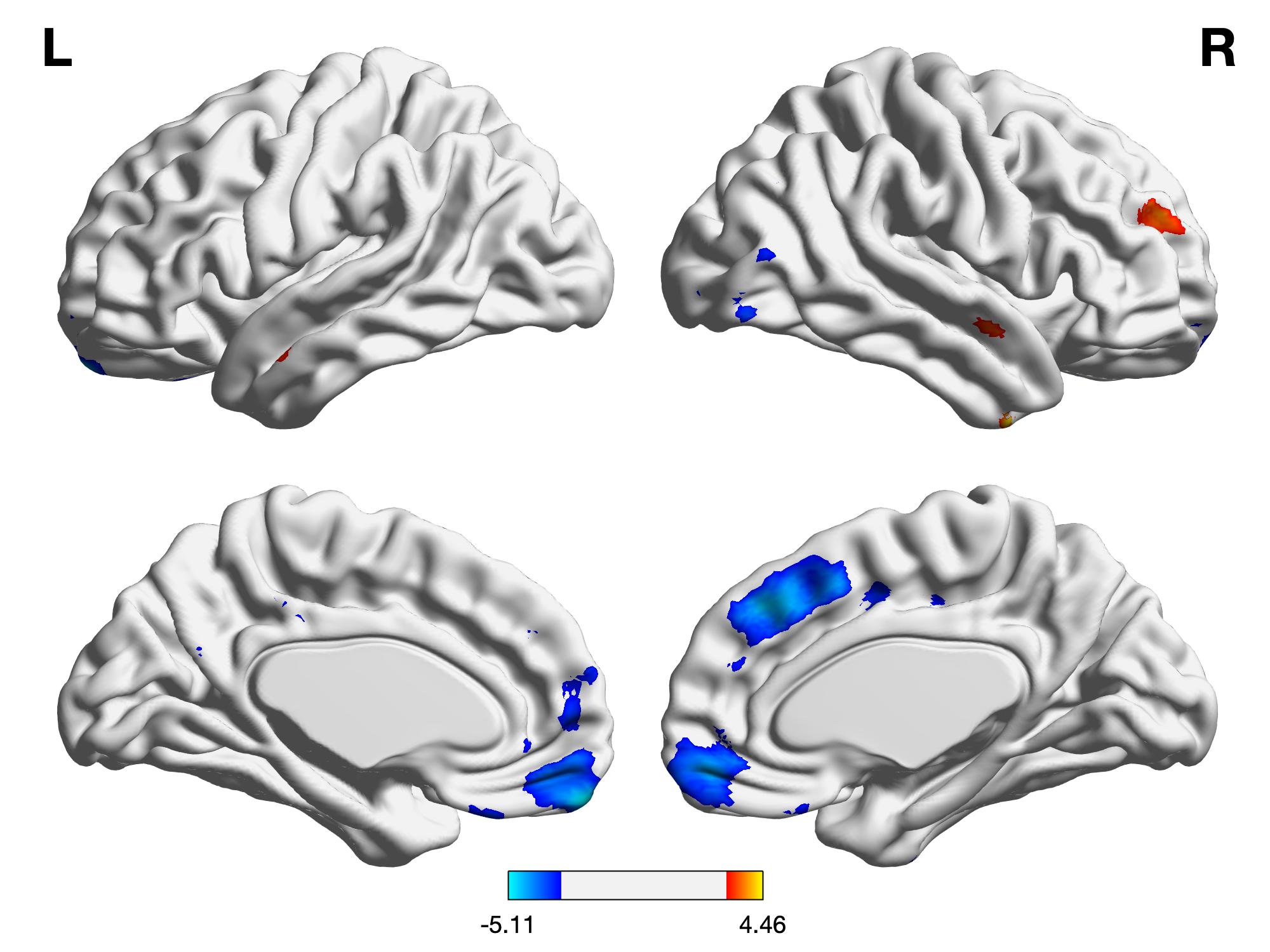


T-map of the correlation with early-life urbanicity and GMV (red means urban > rural, blue means rural > urban, shown at p<0.001 uncorrected).

## Table S1 Regional GMVs that urban subjects are larger than rural subjects

| Peak Region | Cluster Size | x | y | z | T score | |
| --- | --- | --- | --- | --- | --- | --- |
| Middle frontal gyrus | 345 | 30 | 47 | 25 | | 4.22 |
| Temporal pole | 217 | 39 | 6 | -38 | | 4.09 |
| Rolandic operculum | 85 | -48 | 6 | 3 | | 3.74 |
| Cerebellum | 73 | 16 | -37 | -15 | | 3.71 |
| Cerebellum | 49 | -15 | -33 | -17 | | 3.64 |
| Middle temporal gyrus | 14 | 62 | 8 | -26 | | 3.52 |
| Postcentral gyrus | 43 | -34 | -30 | 57 | | 3.48 |
| Middle frontal gyrus | 9 | 44 | 47 | 27 | | 3.34 |
| Middle frontal gyrus | 3 | -27 | 26 | -26 | | 3.24 |
| Inferior frontal gyrus | 1 | 51 | 33 | 28 | | 3.23 |
| Middle frontal gyrus | 2 | 50 | 35 | 30 | | 3.19 |
| Superior frontal gyrus | 2 | 22 | -13 | 67 | | 3.14 |
| Postcentral gyrus | 1 | 30 | -28 | 54 | | 3.14 |
| Temporal pole | 1 | -24 | 6 | -20 | | 3.11 |
| Postcentral gyrus | 1 | 51 | -1 | 30 | | 3.11 |

GMV: Grey Matter Volume

# Supplement References:

Ministry of Housing and Urban-Rural Development, P. R. C. (2014). "China Urban Construction Statistical Yearbook." 66-99.
